# Antioxidant and skin-whitening effects of aerial part of *Euphorbia supina* Raf. Extract

**DOI:** 10.1186/s12906-018-2323-5

**Published:** 2018-09-17

**Authors:** Sa-Haeng Kang, Yong-Deok Jeon, Ji-Yoon Cha, Sung-Woo Hwang, Hoon-Yeon Lee, Min Park, Bo-Ri Lee, Min-Kyoung Shin, Su-Jeong Kim, Sang-Min Shin, Dae-Ki Kim, Jong-Sik Jin, Young-Mi Lee

**Affiliations:** 1 Department of Oriental Pharmacy, College of Pharmacy, Wonkwang-Oriental Medicines Research Institute, Wonkwang University, Iksan, Jeollabuk-do 54538 South Korea; 2 Department of Oriental Medicine Resources, Chonbuk National University, 79 Gobongro, Iksan, Jeollabuk-do 54596 South Korea; 3 Laboratory of YOUCEL, YOUCEL, INC, 78 Iksandaero, Iksan, Jeollabuk-do 54526 South Korea; 4 Department of Immunology and Institute of Medical Science, Jeonbuk National University Medical School, Jeonju, Jeollabuk-do 54896 South Korea

**Keywords:** *Euphorbia supina* (ES), DPPH, Melanogenesis, Tyrosinase, MITF, α-MSH

## Abstract

**Background:**

*Euphorbia supina* (ES) has been widely used in folk medicine owing to its antibacterial, hemostatic, and anti-inflammatory properties. The aim of this study was to evaluate the antioxidant and skin-whitening effects of a 70% ethanol extract of ES.

**Methods:**

The aerial parts of ES plant were extracted with 70% ethanol. The viability of B16F10 cells was evaluated by MTT assay to determine the non-toxic doses for further experiments. The tyrosinase and cellular tyrosinase activities were then measured using an enzyme-substrate assay. In addition, the expression of whitening-related proteins was measured using western blot.

**Results:**

The antioxidant activity of the ES samples increased in a dose-dependent manner, as confirmed by their radical scavenging activities in the 2,2-diphenyl-1-1-picrylhydrazyl and 2,2-azino-bis-(3-ethylbenzthiazoline-6-sulfonic acid) assays. The ES extract significantly reduced tyrosinase activity and melanin content in a dose-dependent manner. Furthermore, it decreased α-melanocyte stimulating hormone (MSH)-induced protein expression of tyrosinase and microphthalmia-associated transcription factor (MITF).

**Conclusions:**

Our results indicate that the ES extract attenuated α-MSH-stimulated melanin synthesis by modulating tyrosinase and MITF expression. Therefore, the ES extract could be a promising therapeutic agent to treat hyperpigmentation and as an ingredient for skin-whitening cosmetics.

## Background

Melanin is a major pigment that controls skin and hair color. It is a high-molecular-weight compound widely distributed in animals and plants [1]. Melanin protects the skin from ultraviolet radiation. However, when produced in excess, the pigment accumulates in the skin to form spots and freckles, and these lesions may cause skin cancer [2]. Therefore, to prevent an excess of skin pigmentation, it is necessary to inhibit the production of melanin [3]. Melanocytes, located in the basal layer of the epidermis and controlled by tyrosinase, produce melanin via melanogenesis, and contain enzymes such as tyrosinase-related protein-1 (TRP-1) and dopachrome tautomerase (DCT) [4]. Tyrosinase catalyzes 3,4-dihydroxyphenylalanine (DOPA) quinone formation from DOPA, and melanin formation from DOPA quinone via autoxidation and enzymatic reactions [5]. Therefore, melanin production is related to tyrosinase expression and TRP-1 activation. Thus, we investigated whether *Euphorbia supina* (ES) could influence the relationship between tyrosinase and melanin.

Tyrosinase activity is important in skin-whitening studies [6]. Various chemicals and plant extracts, such as kojic acid, arbutin, vitamin C, and hydroquinone, are used in skin-whitening cosmetics. However, their use has been limited due to their side effects such as coloration, odor, and cytotoxicity [7]. Therefore, recent studies are focused on the development of skin-whitening agents from skin-safe natural products.

In this study, ES was used to develop a safe compound with antioxidant and skin-whitening effects. ES is an annual herbaceous plant, and is widely used in traditional herbal formulations. It is widely distributed in temperate and tropical regions such as Korea, China, and Japan. It is used in folk medicine against various inflammatory disorders [8, 9]. Studies focused on the components of ES such as tannins [10, 11], phenolic substances and flavonoids [12, 13], and terpenoids [14, 15] have been reported. In addition, the in vitro anticancer effect in breast cancer metastasis [16], and the antioxidant activity of phenolic mixtures of ES have been reported [17]. However, the in vitro antioxidant activities in 2,2-diphenyl-1-1-picrylhydrazyl (DPPH) and 2,2′-azino-bis-3-ethylbenzthiazoline-6-sulfonic acid (ABTS) assays, and skin-whitening effects of ES extracts on melanoma cells have not been understood. Therefore, the aim of this study was to determine the antioxidant and skin-whitening effects of ES in vitro and confirm its efficacy as a novel skin-whitening agent.

## Methods

### Preparation of the ES extract

ES was provided by Wonkwang pharmaceutical company (Iksan, Jeonbuk, Korea). The aerial parts (stem and leaf) of ES (250 g) were boiled with 70% ethanol at 70 °C for 2 h. After filtration, the residue was again boiled twice as described above. The combined filtrates were evaporated at 45 °C and lyophilized to obtain the crude extract at a yield of 60 g (24%). The extract was stored at 4 °C before experiments. A voucher specimen (JUHES-1660) has been deposited at the Department of Oriental Medicine Resources, Chonbuk National University (Iksan, Korea).

### Cell culture

The B16F10 murine melanoma cells (CRL-6475) were purchased from the American Type Culture Collection (ATCC; Manassas, VA, USA). The cells were cultured in Dulbecco’s Modified Eagle’s Medium (DMEM) containing 2 mM glutamine, supplemented with 10% fetal bovine serum (FBS), 100 units/mL of penicillin, and 100 μg/mL of streptomycin in culture flasks in a CO_2_ incubator with a humidified atmosphere of 5% CO_2_ in air at 37 °C. All the experiments were performed in triplicate, and were repeated thrice to ensure reproducibility.

### Cell viability assay

To evaluate the safety of the ES extract, an MTT assay was performed to determine the viability of the cells after treatment with the extract. This method was based on the reduction of 3-(4,5-dimethylthiazol-2-yl)-2,5-diphenyl tetrazolium bromide (MTT) to formazan by mitochondrial enzymes (NAD(P)H-dependent cellular oxidoreductase) in viable cells [18]. Briefly, B16F10 cells (1 × 10^4^ cells/well) were incubated with various concentrations of ES extract (8, 40, 200 μg/mL) in a 96-well microplate for 24 h. Then, the MTT solution (500 μg/mL) was added to each well and the plate was incubated at 37 °C for 8 h. The formazan crystals produced by living cells were dissolved in 600 μL of DMSO. The absorbance of each well was measured at 540 nm on a VersaMax microplate reader (Molecular Devices, Sunnyvale, CA, USA).

### Antioxidant activities

The DPPH radical scavenging activity was evaluated according to the method described in [19] with minor modifications. The various concentration (8, 40, 200 μg/mL) of ES extract or 20 μg/mL of ascorbic acid (used as positive control) at 500 μL were added to 1 mL of 0.1 mM DPPH solution. The reaction was allowed to proceed for 30 min in the dark at room temperature (RT) after vortexing. The absorbance was measured at 517 nm on a VersaMax microplate reader. We conducted an experiment to confirm the extent to which radicals were reduced by the electron-donating effect with respect to the ABTS radical scavenging activity [20]. After radical formation, a solution of 7.4 mM ABTS and 2.6 mM potassium persulfate was added and the resulting mixture was allowed to react for 12 h at RT; ABTS solution was measured and showed an absorbance of 0.70 ± 0.03 (mean ± S.D.) measured using a microplate reader. The ES extract (100 μL) was added to 900 μL of ABTS solution, and was allowed to react for 10 min at RT. The absorbance of 200 μL solution mixture was measured at 732 nm using a microplate reader.

### Measurement of melanin content

Melanin content was measured after adding minor modifications to the previously described protocol [21]. The B16F10 melanoma cells were seeded at 1 × 10^5^ cells/well in 3 mL of medium in 6-well culture plates and incubated overnight to allow cells to adhere. The cells were exposed to various concentrations (8, 40, 200 μg/mL) of the ES extract or 10 mM arbutin for 72 h in the presence or absence of 100 nM of α-melanocyte-stimulating hormone (α-MSH; Sigma-Aldrich, St. Louis, MO, USA). At the end of the treatment, the cells were washed with PBS (pH 7.2) and lysed with 300 μL of 1 M NaOH containing 10% DMSO for 2 h at 80 °C. The absorbance of 200 μL mixture was measured at 400 nm using a microplate reader.

### Determination of cellular tyrosinase activity

Cellular tyrosinase activity was measured according to a method previously described by Lee et al. [22] with some modifications. Six-well plates containing 2 mL of DMEM were seeded with B16F10 melanoma cells at a density of 1 × 10^5^ cells/well. Each well was separated into 6 different groups as follows; Vehicle: non-treatment, Control: 100 nM of α-MSH, ESEE 8: α-MSH and ES extract (8 μg/mL), ESEE 40: α-MSH and ES extract (40 μg/mL), ESEE 200: α-MSH and ES extract (200 μg/mL), Arbutin: α-MSH and arbutin (10 mM). Plates were incubated overnight in a humidified incubator at 37 °C with 5% CO_2_ atmosphere to allow cells to adhere. Cells were then exposed to 100 nM of α-MSH for 48 h and then treated with increasing doses of the ES extract or arbutin for 24 h. The cells were washed with PBS (pH 6.8) and re-suspended in lysis buffer. Next, the cells were ruptured by freezing and thawing, and the lysate was clarified by centrifugation at 16,000 g for 20 min. The protein content of the lysate was determined using a BCA Protein Assay Kit (Thermo Scientific, Vantaa, Finland). After quantifying the protein levels, the concentrations were adjusted such that all the samples contained the same amount of protein (20 μg). These lysates were then added to the wells of a 96-well plate containing 2.5 mM L-DOPA in 0.1 M phosphate buffer (pH 6.8). Following incubation at 37 °C for 1 h, the absorbance of the different lysates was measured at 475 nm using a spectrophotometer. Tyrosinase activity in the protein was calculated by the following formula:

$$ {\displaystyle \begin{array}{c}\mathrm{Tyrosinase}\ \mathrm{activity}\ \left(\%\right)=\left({\mathrm{OD}}_{\mathrm{s}}-{\mathrm{OD}}_{\mathrm{b}}\right)/\mathrm{Control}\times 100\\ {}{\mathrm{OD}}_{\mathrm{s}}:\mathrm{sample}\ \mathrm{absorbance}\ \mathrm{value}\\ {}{\mathrm{OD}}_{\mathrm{b}}:\mathrm{vehicle}\ \mathrm{absorbance}\ \mathrm{value}\end{array}} $$

### Mushroom tyrosinase activity

L-DOPA was used as the substrate for the measurement of mushroom tyrosinase activity. The reaction buffer (total 3 mL) used in the experiment contained 2.8 mL of 0.5 mM L-DOPA in 50 mM Na_2_HPO_4_-NaH_2_PO_4_ buffer (pH 6.8) and 100 μL of different concentrations of ES. An aqueous solution of mushroom tyrosinase (100 μL) was added to the mixed buffer. The solution (200 μL) was immediately evaluated for the linear increase in optical absorbance at 475 nm using a microplate reader.

### Western blot analysis

The B16F10 melanoma cells (1 × 10^6^ cells/well) were cultured in 60-mm^2^ dishes. Then, cells were treated with various concentrations (8, 40, 200 μg/mL) of ES extract and 10 mM of arbutin for 24 h. The cells were then lysed in a buffer containing 50 mM Tris-HCl with pH 7.4, 2 mM EGTA, 1 mM phenylmethyl-sulfonyl fluoride, 10 mM β-glycerophosphate, 10 mM β-mercaptoethanol, 1 mM sodium orthovanadate, and 0.1% deoxycholic acid sodium salt. The lysates (25 μg) were resolved by 10% SDS-polyacrylamide gel electrophoresis and transferred electrophoretically to polyvinylidene difluoride membranes and blocked overnight with 5% skim milk in TBST buffer (20 mM Tris-HCl pH 7.4, 100 mM NaCl, and 0.1% Tween 20) at 4 °C. After the membranes were washed in TBST buffer, they were incubated for 3 h with a primary antibody of tyrosinase, MITF, and β-actin (ratio 1:1000). After incubation with a secondary antibody (ratio 1:1000) for 1 h at RT, the protein-antibody complexes were visualized with ECL Western blotting Luminol Reagent (Santa Cruz Biotech, CA, USA), and detected using the Fluorchem E image analyzer (Cell Biosciences, CA, USA).

### GC-MS analysis

The ethanol extract sample was dried with a speed vacuum and derivatized with *N*,*O*-bis(trimethylsilyl)trifluoroacetamide (BSTFA) (Sigma Aldrich, St. Louis, MO, USA). GC-MS analysis was performed using a QP2010 gas chromatograph coupled with a mass spectrometer (Shimadzu) at an ionization voltage of 70 eV. GC analysis was performed in a temperature-programming mode with a Restek column (0.25 mm, 30 m; XTI-5). The initial column temperature was set at 70 °C for 3 min, increased linearly at 10 °C/min to 300 °C, and then held for 5 min. The temperature of the injection port was 280 °C, and the GC/MS interface was maintained at 290 °C. The helium carrier gas flow rate was 1.0 mL/min.

### Statistical analysis

The data for melanin synthesis, cytotoxicity, and tyrosinase activity assay were statistically evaluated by ANOVA, followed by Dunnett’s test. The data are presented as mean ± SD. A *P* value < 0.05 indicated statistical significance.

## Results

### Effect of the ES extract on B16F10 cell viability

**Fig. 1:**
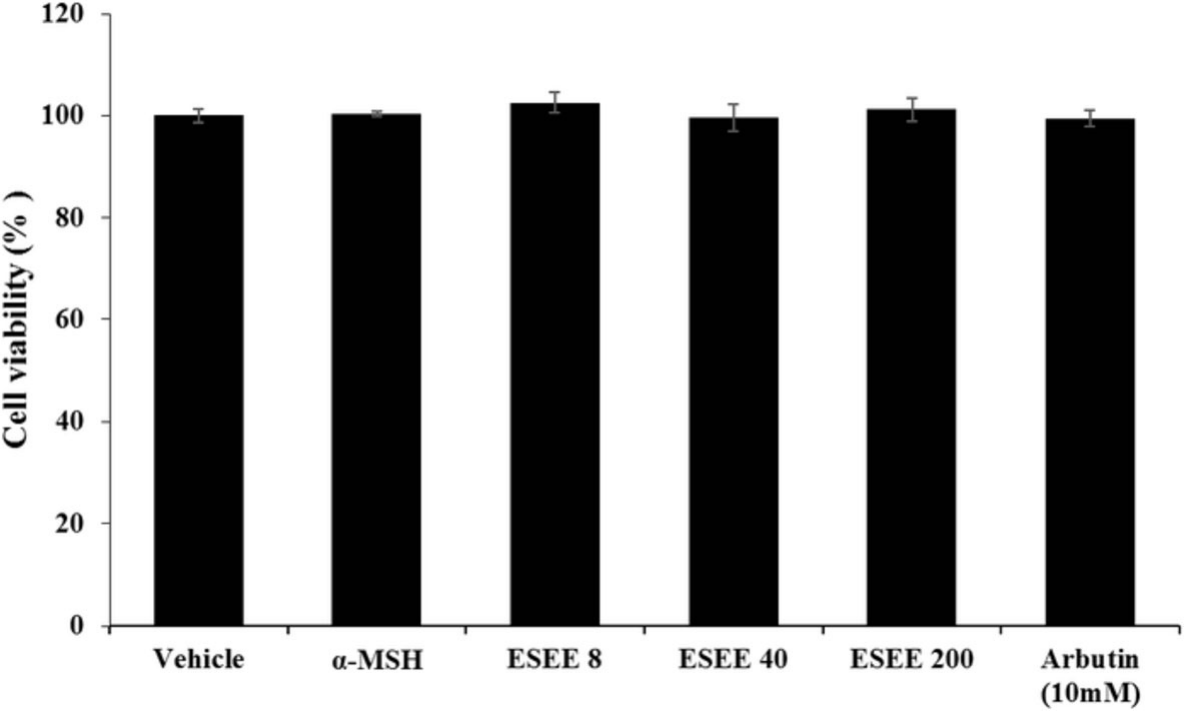
Cell viability of the ES extract on the B16F10 cells. B16F10 cells were treated with various concentration of ES extract (8, 40, 200 μg/ml) for 24 h and the cell viability was measured by MTT assay. The absorbance was measured at 540 nm on a VersaMax microplate reader. Values represent the mean ± S.D. of triplicate experiments

The MTT assay was used to assess the effect of the ES 70% ethanol extract on the viability of B16F10 melanoma cells. The cells were treated with various concentrations of the extract (8, 40, 200 μg /mL) for 24 h, and then the MTT assay was performed. The results are expressed as percent of viability relative to control. The ES extract had a non-cytotoxic effect on B16F10 cell proliferation (Fig. 1). However, at a concentration of 1000 μg/mL, clear cytotoxicity was observed in B16F10 cells (data not shown).

### Antioxidant capacities of the ES extract

**Fig. 2:**
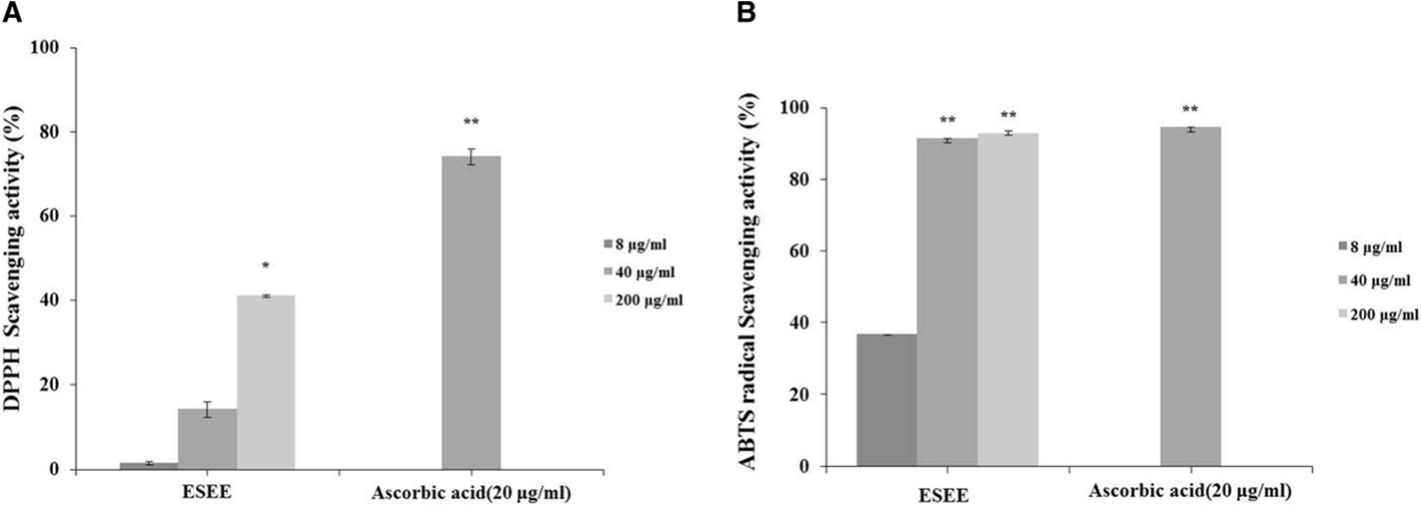
Antioxidant activities of ES extract. **a** Scavenging effect of ES on DPPH radical (**b**) ABTS^+^ radical scavenging activity of the extract. The ES ethanol extract (ESEE) (8, 40, 200 μg/ml), ascorbic acid (20 μg/ml) were incubated with DPPH, ABTS^+^ solution, respectively. Results are represented as percentages of control, and the data represent mean ± S.D. for three separate experiments. Values are significantly different by comparison with control. *: *p* < 0.05, **: *p* < 0.01

The DPPH and ABTS assays were used to measure the antioxidant activity of the ES extract. To measure the DPPH radical scavenging activity of the ES extract, various concentrations of the ES extract (8, 40, 200 μg/mL) were used. The DPPH radical scavenging activity of the ES extract increased in a dose-dependent manner. Ascorbic acid used as a positive control and showed over 50% DPPH radical scavenging activity (Fig. 2a). The ABTS^+^ radical scavenging activity of 8 and 40 μg/mL of ES extract was also significantly high, presenting a result similar to the positive control group. The ES extract showed 93.05 ± 0.6% activity at 200 μg/mL, which was almost the same as that of ascorbic acid (Fig. 2b).

### Effect of the ES extract on mushroom tyrosinase activity, B16F10 melanin content, and intracellular tyrosinase activity

**Fig. 3:**
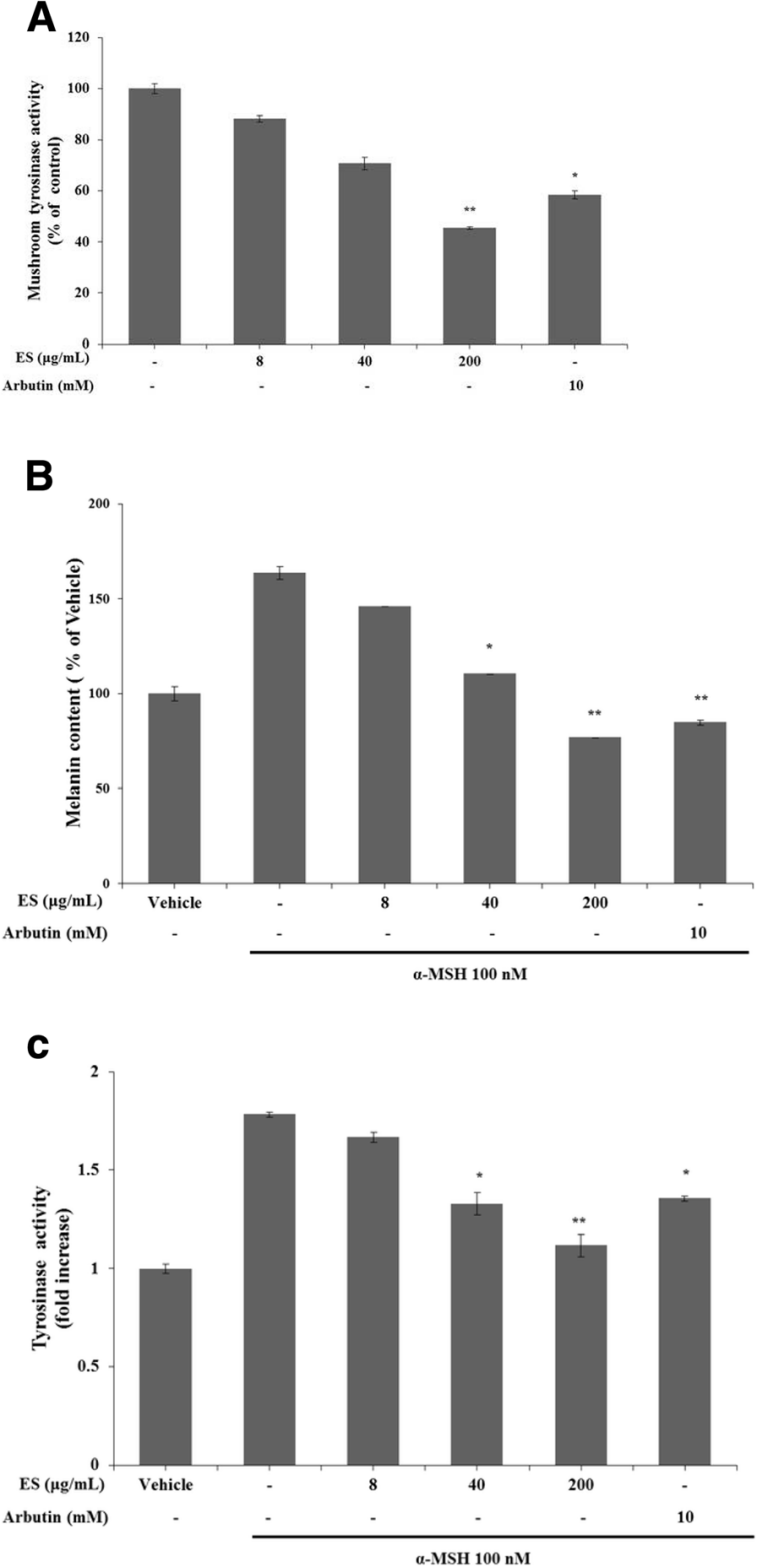
Inhibitory effect of ES extract on mushroom tyrosinase activity, B16F10 melanin content and intracellular tyrosinase activity. **a** Different concentrations of the ES extract (8, 40, 200 μg/ml) or arbutin (10 mM) was incubated with the same units of mushroom tyrosinase. Following incubation, the amount of dopachrome produced was determined at 490 nm. **b** and **c** B16F10 melanoma cells were stimulated with α-MSH (100 nM) for 48 h, and then the melanin content or intracellular tyrosinase activity were measured after treatment with various concentrations of the ES extract (8, 40,200 μg/ml) or arbutin (10 mM) for another 24 h. Results are represented as percentages of control, and data are presented as mean ± S.D. for three separate experiments. Values are significantly different by comparison with control. *: *p* < 0.05, **: *p* < 0.01 α-MSH treatment alone

To measure the inhibitory effect of the ES extract on mushroom tyrosinase activity, the tyrosinase inhibition assay was performed. The results indicated that the mushroom tyrosinase activity was inhibited by the ES extract at high concentrations. Tyrosinase activity was 88.35 ± 1.28%, 70.81 ± 2.52%, and 45.43 ± 0.48% after treatment with 8, 40, and 200 μg/mL of the ES extract, respectively (Fig. 3a). To examine the antimelanogenic activity of the ES extract, the inhibitory effect of the ES extract on melanin was evaluated in B16F10 cells. The B16F10 cells were treated with the ES extract at 8, 40, and 200 μg/mL or with arbutin at 10 mM, and stimulated with α-MSH (10 nM) for 48 h. The ES extract presented a significant dose-dependent inhibitory effect on melanin synthesis. The melanin content was represented as percentage of the vehicle. The melanin content was 146.17 ± 0.07%, 110 ± 0.1%, and 76.95 ± 0.39% after treatment with 8, 40, and 200 μg/mL of the ES extract, respectively (Fig. 3b). When the B16F10 cells were treated with the positive control arbutin (10 mM), the intracellular melanin content was 84.82 ± 1.28%. To determine the mechanism underlying the inhibitory effect of the ES extract on melanogenesis, we assessed the intracellular tyrosinase activity in B16F10 melanoma cells. Therefore, another group of B16F10 cells was treated with various concentrations of the ES extract (8, 40, 200 μg/mL) or arbutin (10 mM) and stimulated with α-MSH (10 nM) for 48 h. The ES extract significantly inhibited α-MSH-induced tyrosinase activity in a dose-dependent manner (Fig. 3c).

### Inhibitory effect of the ES extract on proteins related to melanogenesis in B16F10 cells

**Fig. 4:**
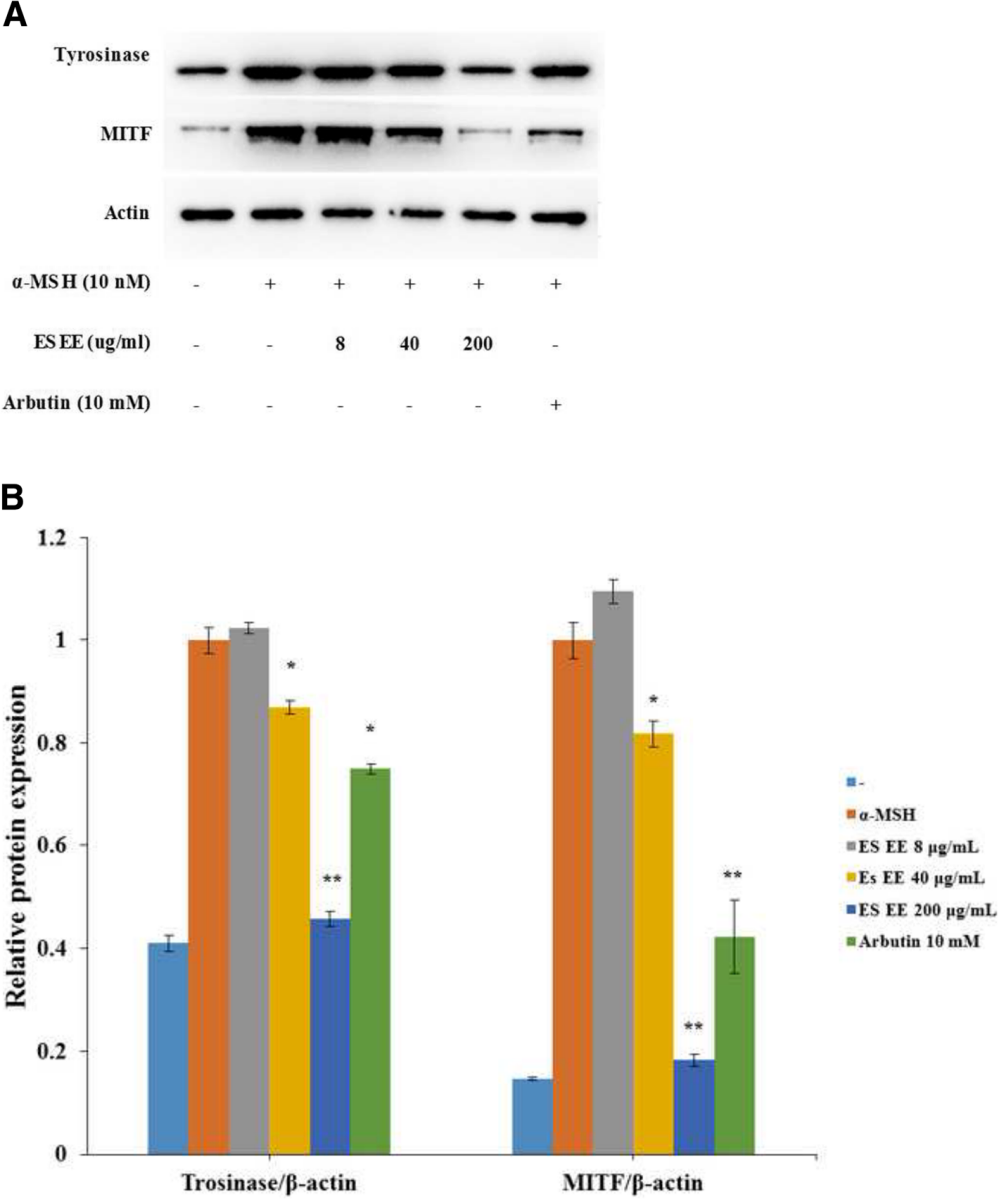
Effect of ES extract on melanogenesis-related proteins expression. **a** B16F10 cells were cultured with α-MSH (100 nM) for 24 h and then treated with various concentration of the ES extract (8, 40, 200 μg/ml) or arbutin (10 mM) for another 24 h. Then the content of cellular MITF, tyrosinase proteins were analyzed by western blotting assay. **b** Densitometry was normalized to the α-MSH group. Values are mean ± S.D. Data were analyzed by Student’s t-test. *: *p* < 0.05, **: *p* < 0.01 versus α-MSH treatment alone

To investigate whether ES could influence melanogenic protein expression, western blotting was performed using the lysate of B16F10 melanoma cells treated with ES and stimulated with α-MSH (10 mM). Tyrosinase and MITF expression levels were evaluated by western blot (Fig. 4). We confirmed that tyrosinase and MITF expression significantly increased when cells were stimulated with α-MSH. The ES extract was able to suppress tyrosinase and MITF expression stimulated by α-MSH in a dose-dependent manner.

### Chemical composition of ES

**Fig. 5:**
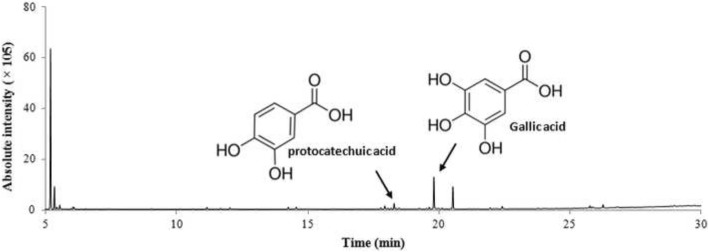
GC-MS Chromatograms of the methanol extract ES

The chemical composition of the ES extract was analyzed by GC-MS (Fig. 5). The retention times of gallic and protocatechuic acids, two of the major components of the ES extract, were 19.810 and 18.290 min, respectively. The two compounds were successfully detected in the ES extract.

## Discussion

In this study, we evaluated whether the ES extract exhibited a skin-whitening effect by suppressing melanin production in B16F10 melanoma cells. The ES extract inhibited tyrosinase enzyme activity, decreased melanin content, and showed an antioxidant effect. Melanin production may be stimulated by UV or α-MSH. The α-MSH binds MC1R and activates the signaling protein adenylate cyclase to increase cyclic AMP (cAMP) production [23]. cAMP responsive element binding protein (CREB) is activated continuously by protein kinase A (PKA), and activation by CREB increases the expression of MITF protein and promotes the expression of tyrosinase [24]. After stimulating B16F10 cells with α-MSH to induce melanogenesis, the whitening effect of ES was confirmed by melanin quantification. Melanin content was found to be suppressed in a dose-dependent manner, and at the highest concentration (200 μg/mL) used in the experiment, more than 50% suppression of the melanin content was observed in comparison with the α-MSH only treatment group.

ES has been used in traditional herbal formulations. It belongs to the Euphorbiaceae family, and is traditionally used in eastern Asia for medicinal purposes. ES contains numerous biologically active substances including tannins, terpenoids, and polyphenols [25]. DPPH is a relatively stable free radical. The DPPH assay is widely used to measure the antioxidant activity of plants, indicated by a decrease in the deep purple color of the solution by reducing agents such as ascorbic acid, tocopherol, polyhydroxy aromatic compounds, and aromatic amines [26]. In addition, ABTS radical scavenging activity is more useful than DPPH radical scavenging activity with respect to measurement of hydrogen-donating antioxidants, chain-breaking antioxidants, and antioxidant ability of hydrophilic and hydrophobic substances [27]. Studies have reported the antioxidant activity of ES. However, its skin-whitening effect has not been evaluated thus far. Therefore, in this study, we confirmed the skin-whitening effect of ES (Fig. 2). We verified its influence on tyrosinase activity, which determines the initial rate of melanogenesis (Fig. 3). As an enzyme that promotes the production of melanin, tyrosinase is widely used as a target compound to identify new inhibitors that can suppress melanin formation [28]. To investigate the mechanism by which ES inhibits melanogenesis, we determined whether the ES extract could inhibit tyrosinase activity directly [29]. For this assay, arbutin was used as the positive control. We observed a skin-whitening effect, which was remarkably higher than that of arbutin (10 mM). The ES extract significantly decreased the mushroom tyrosinase activity after 24 h of treatment and inhibited cellular tyrosinase activity. Thus, we confirmed that the ES extract exhibits an excellent antioxidant effect as well as skin-whitening activity.

Tyrosinase is the main enzyme involved in melanogenesis [30]. The MITF transcription factors are important regulators in the development and differentiation of melanocytes [31]. The suppression of tyrosinase and MITF expression by the ES extract increased in a dose-dependent manner. These results suggest that ES suppresses the transcription factor MITF, and thus inhibits tyrosinase expression.

There has been an increase in the interest in various natural products with skin-whitening and wrinkle-improvement effects as base materials for food and cosmetics. The use of products containing ingredients with various skin-protecting properties, such an anti-wrinkle, moisturizing, whitening, and anti-inflammation, has increased in the global beauty market [32]. In this study, we found that the ES extract had strong antioxidant effects, inhibited tyrosinase, and regulated whitening-related protein expression. The activities might be attributed to gallic acid and protocatechuic acid detected in ES extract [33, 34]. Therefore, ES appears to have the potential to be used for the development of natural cosmetics for skin whitening for effective reduction or prevention of excessive melanin pigmentation in the skin.

## Conclusions

In conclusion, ES extract regulated tyrosinase activities and MITF protein expression in B16F10 cells. The ES extract showed antioxidant activities in DPPH and ABTS assays. This study provides experimental evidence that ES could be a useful therapeutic agent for the treatment of skin diseases.

## Abbreviations

ABTS: 2,2′-azino-bis-3-ethylbenzthiazoline-6-sulfonic acid
cAMP: Cyclic AMP
CREB: cAMP responsive element binding protein
DCT: Dopachrome tautomerase
DMEM: Dulbecco’s Modified Eagle’s Medium
DPPH: 2,2-Diphenyl-1-1-picrylhydrazyl
ES: *Euphorbia supina*
FBS: Fetal bovine serum
MITF: Microphthalmia-associated transcription factor
MTT: 3-(4,5-dimethylthiazol-2-yl)-2,5-diphenyl tetrazolium bromide
PKA: Protein kinase A
RT: Room temperature
TRP-1: Tyrosinase-related protein-1
α-MSH: α-melanocyte-stimulating hormone
